# Supplementary material for: A new STI in the city: MPOX in Barcelona. First outbreak (5/2022-5/2023) and subsequent resurgence
Source: PLoS One. 2025 Jan 16;20(1):e0296141. doi: 10.1371/journal.pone.0296141 (PMC11737761; doi:10.1371/journal.pone.0296141)
Supplement: S1 Appendix — Posters displayed at local saunas and sex clubs during August 2022. (DOCX) [file pone.0296141.s001.docx]

**S1. Appendix.**

Printed Poster.

*English translation after the image. Certain images and logos have been obscured to comply with copyright requirements and the Creative Commons Attribution 4.0 International (CC BY 4.0) licenes.*

**
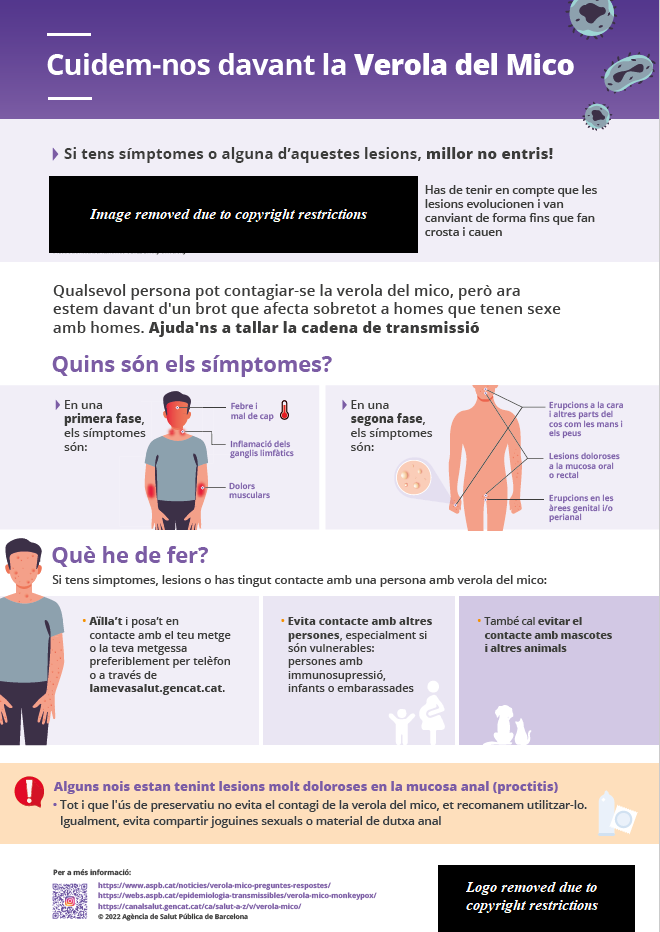
**

***Let’s Take Precautions Against MPOX***

*If you have symptoms or notice any lesions, please avoid entering! These lesions may progress and eventually scab over. While anyone can contract MPOX, the current outbreak primarily affects men who have sex with men. Let’s work together to break the chain of transmission.*

***What are the symptoms?***

*In the early stages, symptoms may include fever, headache, swollen lymph nodes, and muscle pain. Later, a rash can appear on the face and other parts of the body, along with painful lesions on the oral or rectal mucosa, and genital or perianal areas.*

***What should you do?***

*Isolate yourself and contact your doctor, ideally by phone or through a healthcare app.*

*Avoid contact with others, especially vulnerable individuals.*

*Prevent contact with pets or other animals.*

***Important Note:***

*Some individuals are experiencing severe pain from rectal mucosa lesions (proctitis). While condoms do not fully prevent the spread of the disease, their use may still be advisable. Similarly, avoid sharing sex toys or douching equipment.*
