# Supplementary material for: A new STI in the city: MPOX in Barcelona. First outbreak (5/2022-5/2023) and subsequent resurgence
Source: PLoS One. 2025 Jan 16;20(1):e0296141. doi: 10.1371/journal.pone.0296141 (PMC11737761; doi:10.1371/journal.pone.0296141)
Supplement: S2 Appendix — Infographics published in the Instagram account @provesrapides_aspb. (DOCX) [file pone.0296141.s002.docx]

**S2. Appendix.**

Social media carrousel infographics.

*Certain images and logos have been obscured to comply with copyright requirements and the Creative Commons Attribution 4.0 International (CC BY 4.0) licenes Complete info available at Instagram: @provesrapides_aspb).*

1. **Stigma vs. health**


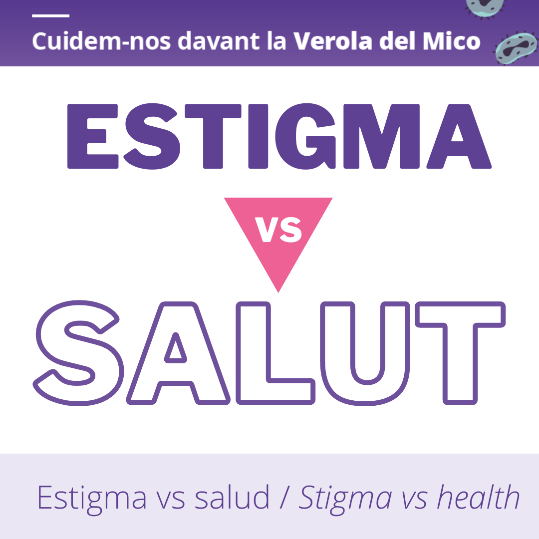

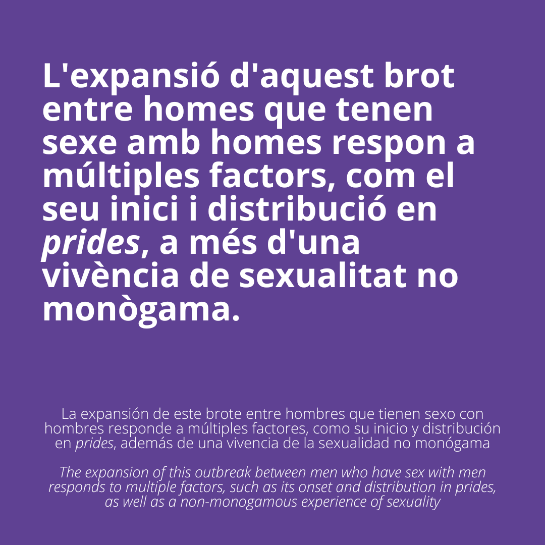

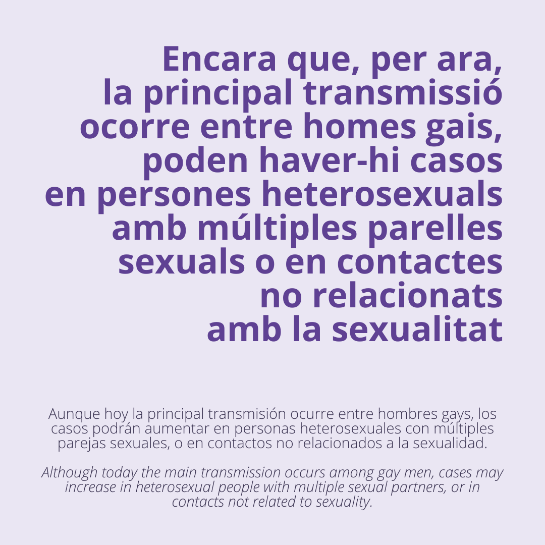

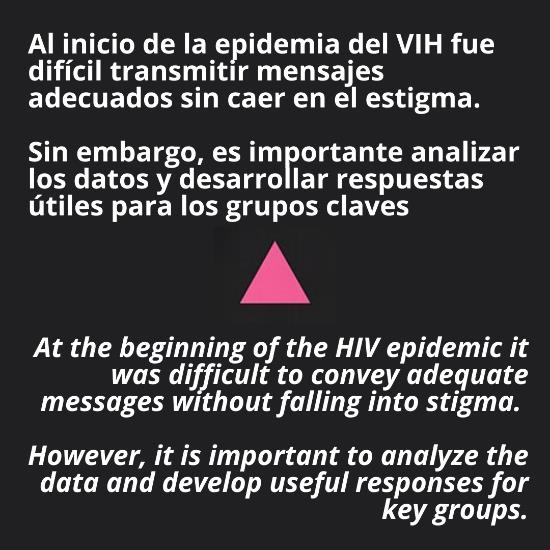

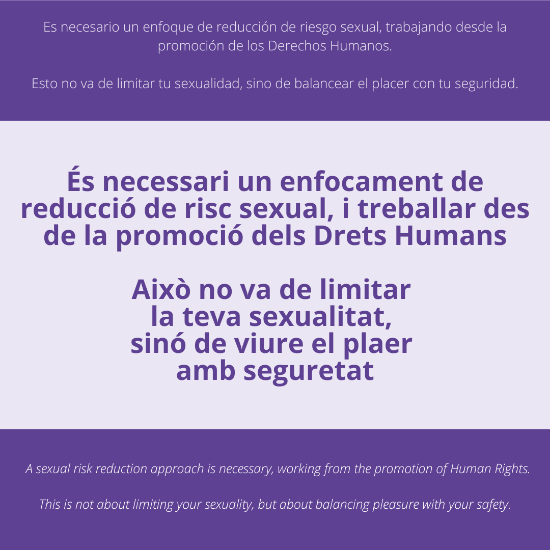


1. **Should I decrease the number of sex partner**


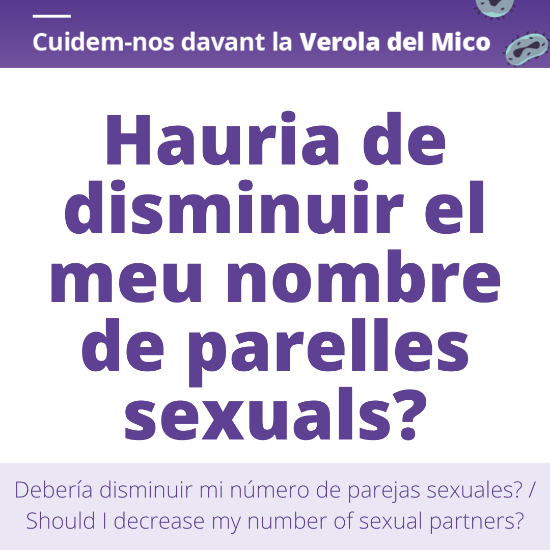

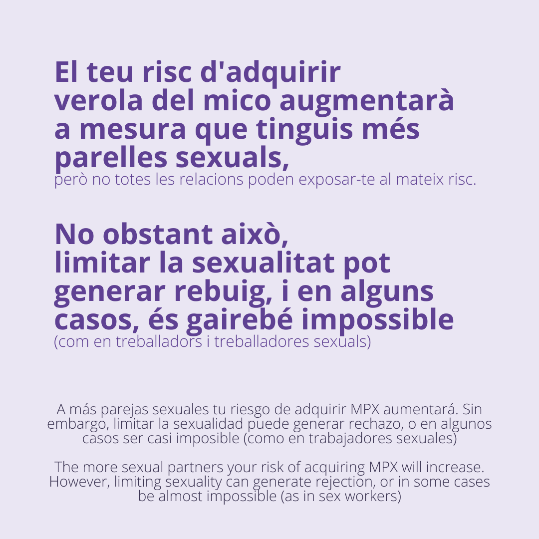

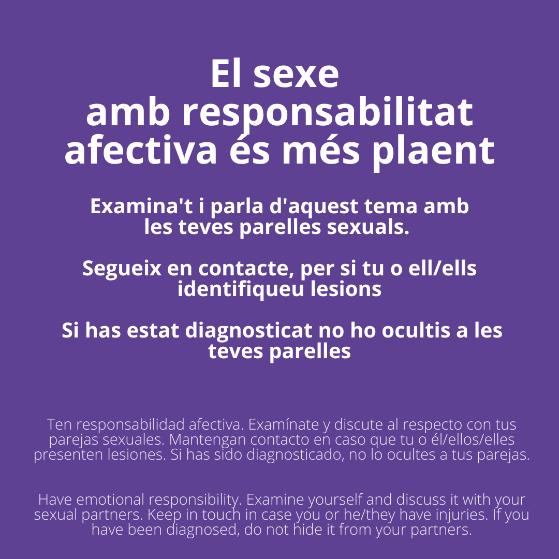

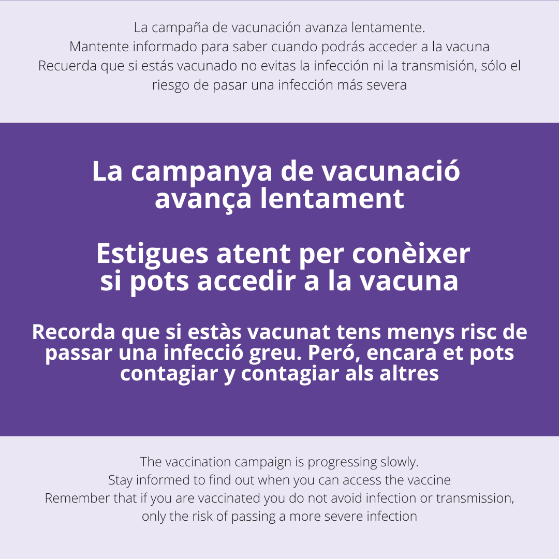

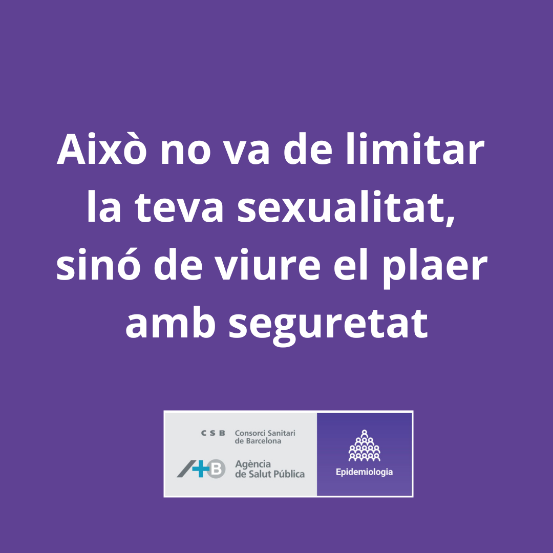


***Logo removed due to copyright restrictions***

1. **What to do if:**


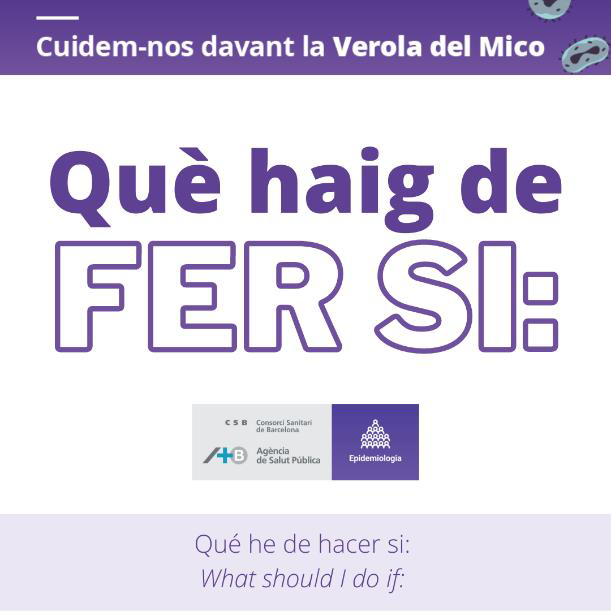

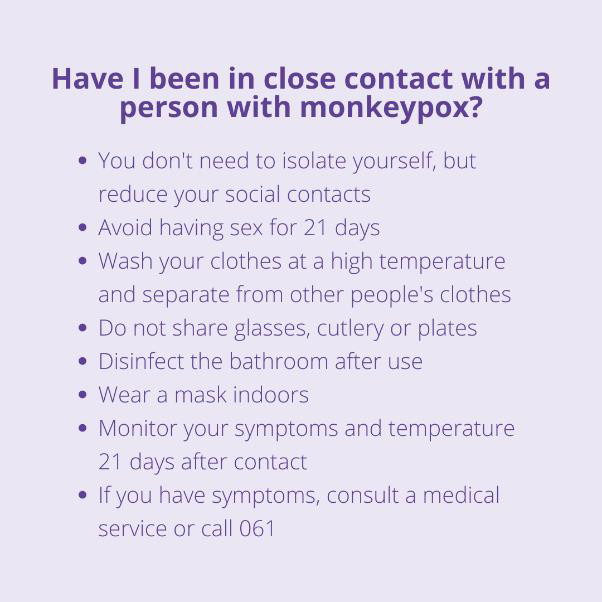

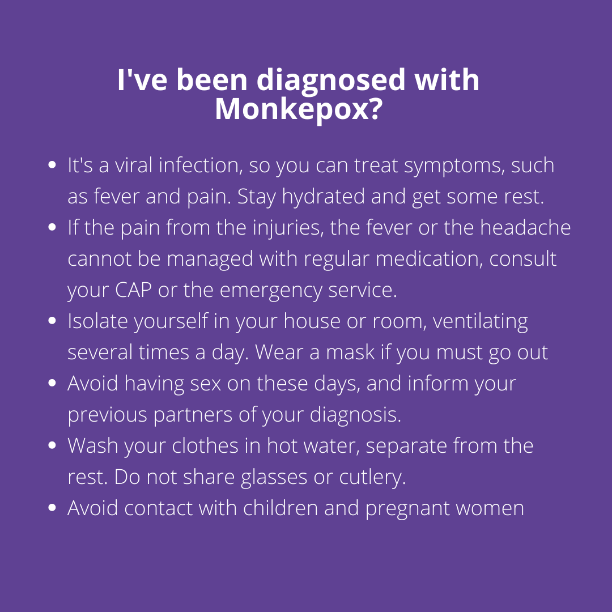

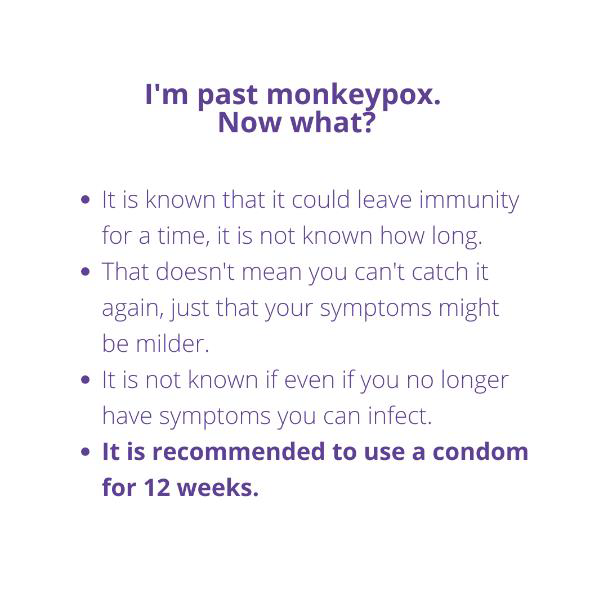


***Logo removed due to copyright restrictions***

1. **Is the condom effective in reducing transmission?**


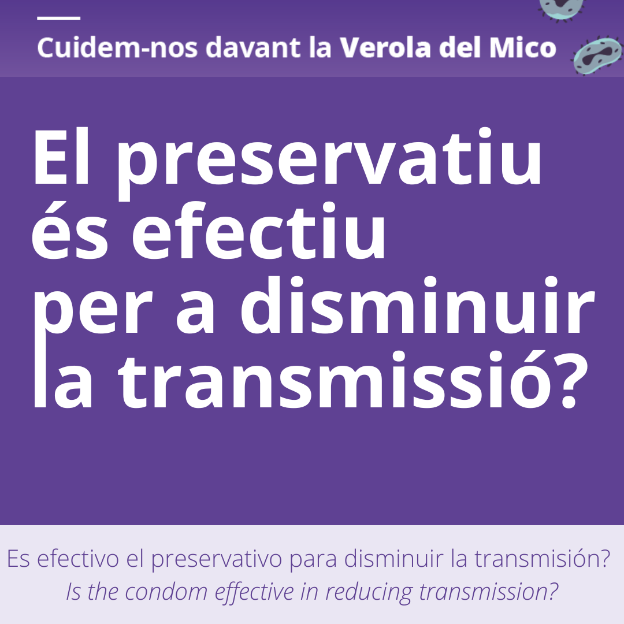

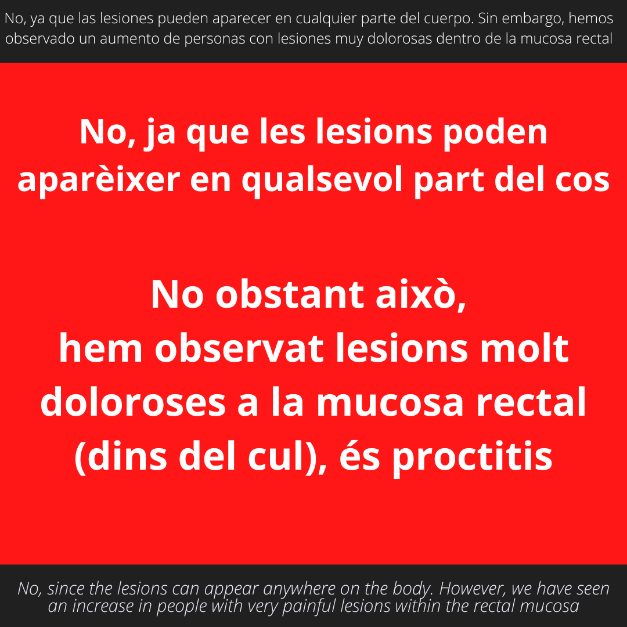

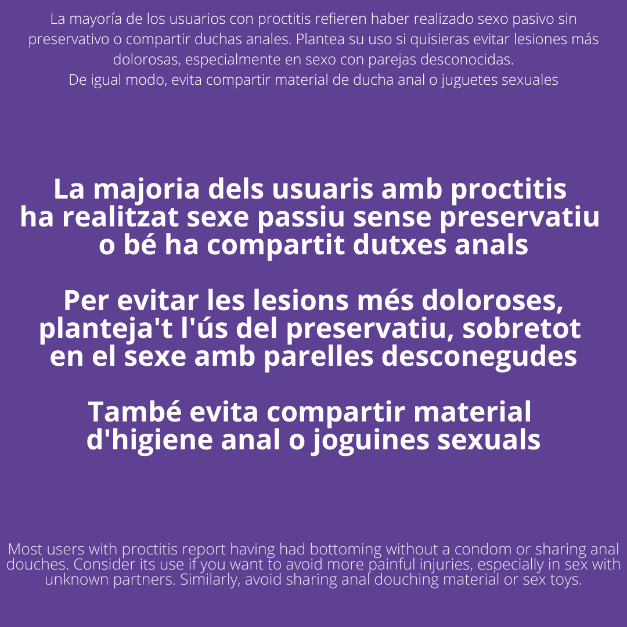

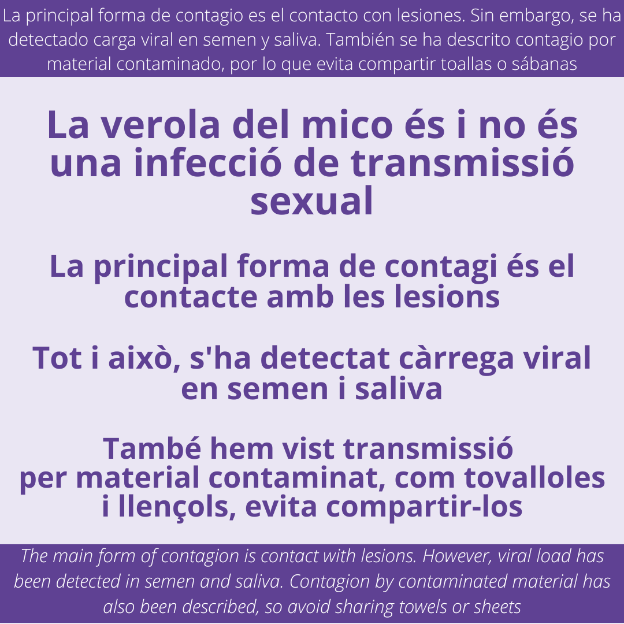


1. **Monkeypox or a pimple?**


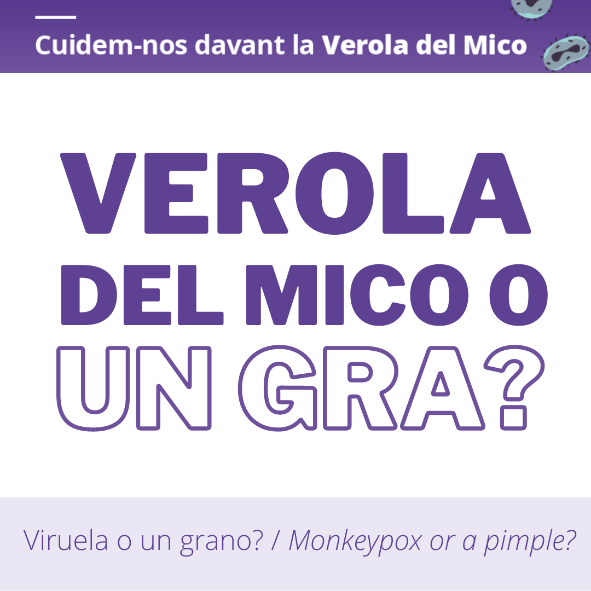

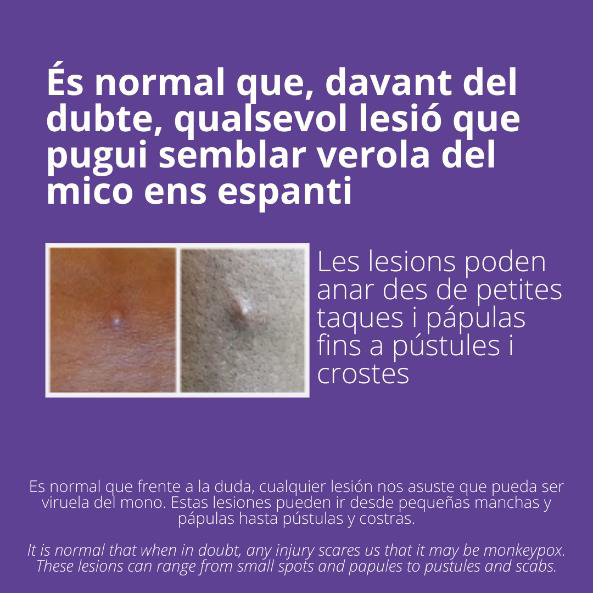

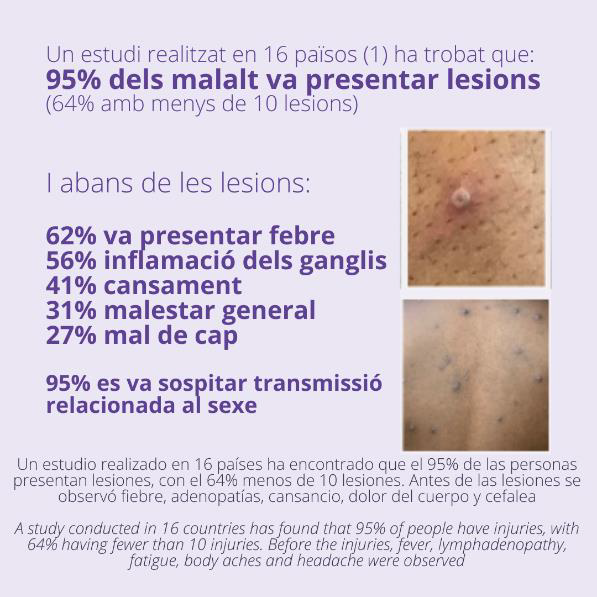

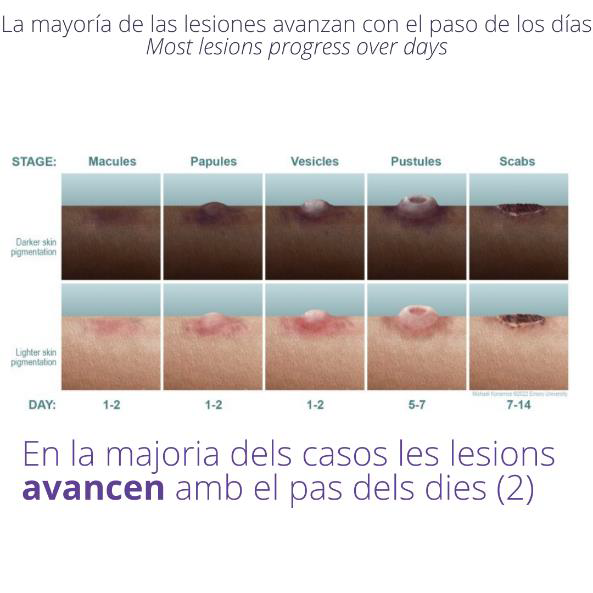

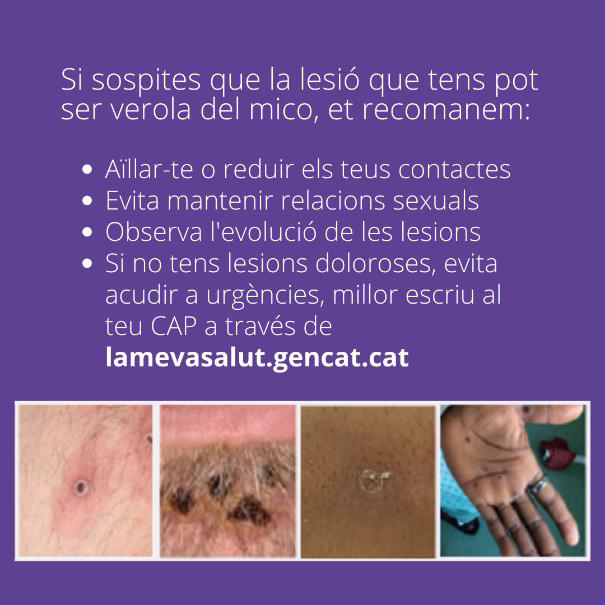

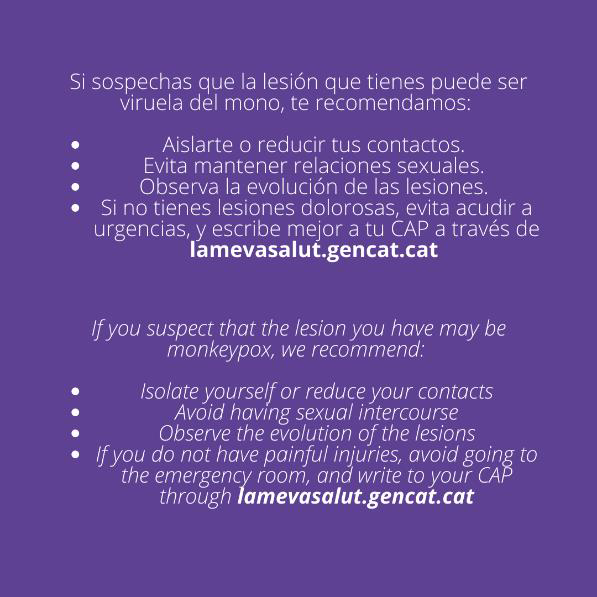


***Image removed due to copyright restrictions***

***Image removed due to copyright restrictions***

***Image removed due to copyright restrictions***

1. **Will I get MPOX if I go to a summer festival geared toward gay men?**


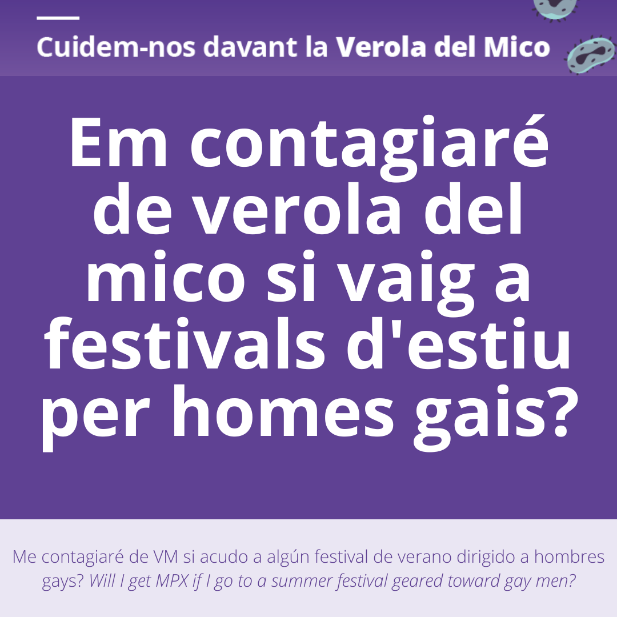

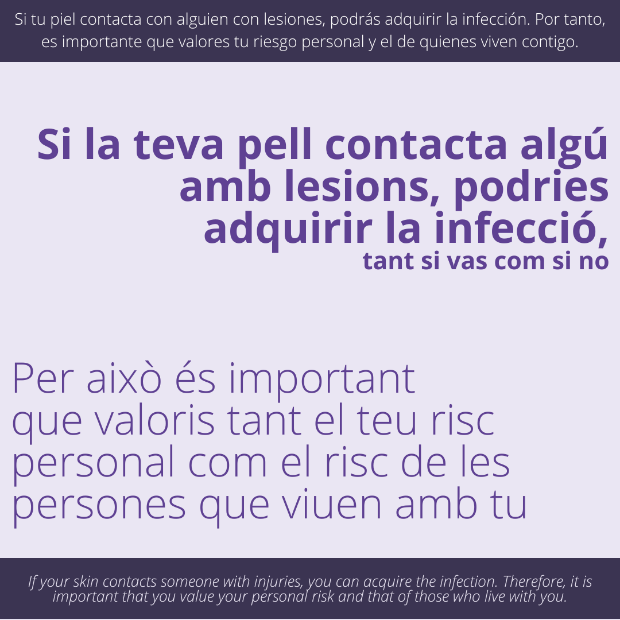

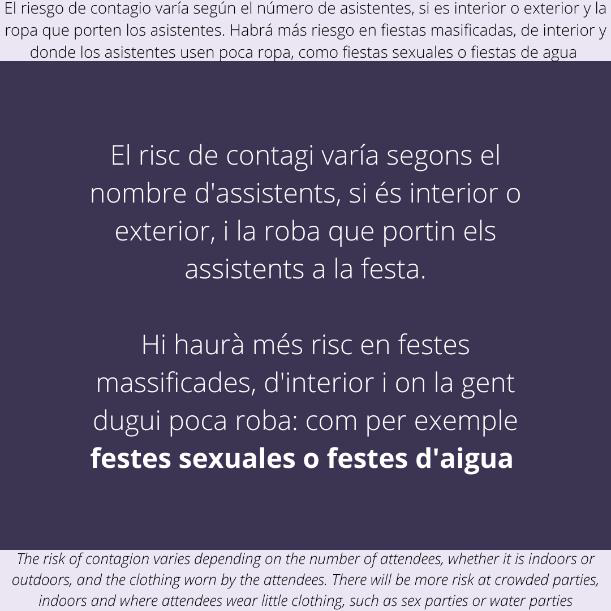

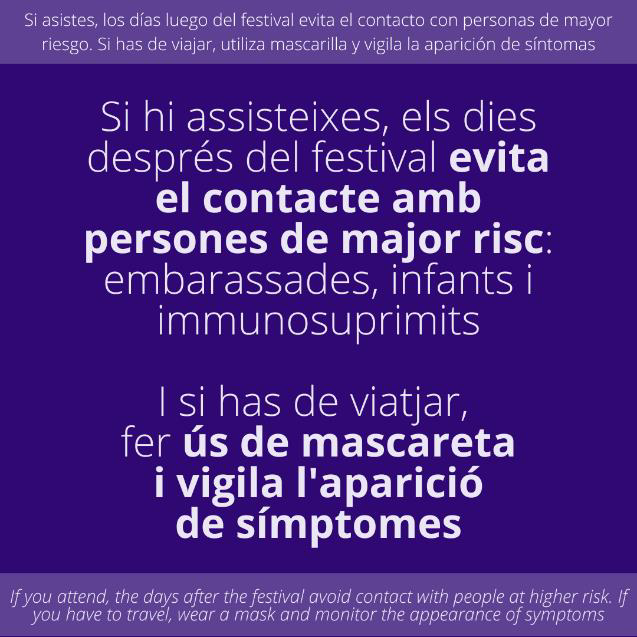

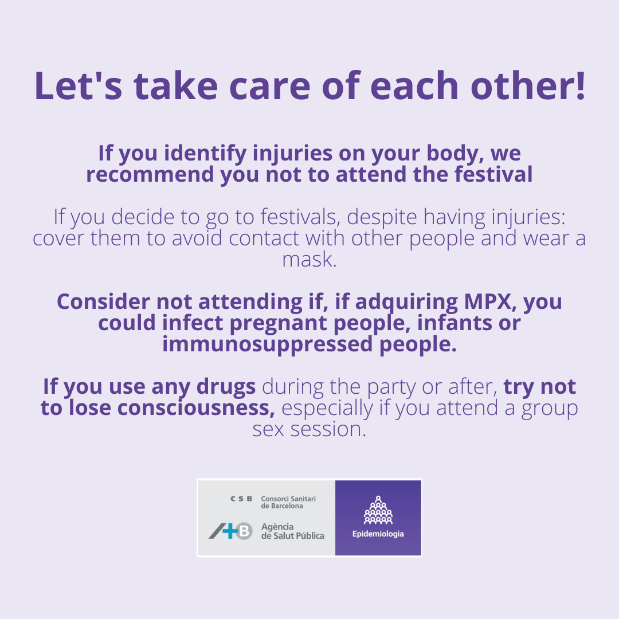


***Logo removed due to copyright restrictions***
