# Supplementary material for: A new STI in the city: MPOX in Barcelona. First outbreak (5/2022-5/2023) and subsequent resurgence
Source: PLoS One. 2025 Jan 16;20(1):e0296141. doi: 10.1371/journal.pone.0296141 (PMC11737761; doi:10.1371/journal.pone.0296141)
Supplement: Attachment — Submitted filename: Letter to editors - MPOX PONE.pdf [file pone.0296141.s003.pdf]

Rebuttal Letter in response to the editor:

Barcelona, August 10, 2023

Dear editor Dr. Milad Zandi:

We sincerely appreciate the revision of our manuscript and all the improvements that could be made. We will respond to the comments made and hope to advance in our proposal.

*1. If applicable, **we recommend that you deposit your laboratory protocols** in protocols.io to enhance the reproducibility of your results. Protocols.io assigns your protocol its own identifier (DOI) so that it can be cited independently in the future. For instructions see: https://journals.plos.org/plosone/s/submission-guidelines#loc-laboratory-protocols. Additionally, PLOS ONE offers an option for publishing peer-reviewed Lab Protocol articles, which describe protocols hosted on protocols.io.*

We have discussed your recommendation, but we have decided that it is not a necessity, as it has already been published. The laboratory testing presented in this manuscript is extensively described in the national and regional guidelines of Spain and Catalonia (references 14 and 15). Therefore, we have not deposited the laboratory protocol as it is not necessary for the replication of our study. We have clarified this information in the corresponding section of the manuscript: Lines 140-141: “Following the guidelines of the national and regional protocols of Spain (15) and Catalonia (15), during the month of May 2022...”

*2. Please ensure that your manuscript meets PLOS ONE's **style requirements,** including those for file naming. The PLOS ONE style templates can be found at https://journals.plos.org/plosone/s/file?id=wjVg/PLOSOne_formatting_sample_main_body.pdf and https://journals.plos.org/plosone/s/file?id=ba62/PLOSOne_formatting_sample_title_authors_affiliations.pdf*

We have taken note of all the formatting indications. While we tried to comply with all the regulations in the first submission, we noticed that some affiliations could be improved, as well as the reference style (brackets instead of parentheses). The revised manuscript now fully adheres to the formatting guidelines.

*2. We note that you have indicated that there are restrictions to data sharing for this study. PLOS only allows data to be available upon request if there are **legal or ethical restrictions on sharing data publicly**. For more information on unacceptable data access restrictions, please see http://journals.plos.org/plosone/s/data-availability#loc-unacceptable-data-access-restrictions.*

Before we proceed with your manuscript, please address the following prompts:

a) If there are ethical or legal restrictions on sharing a de-identified data set, please explain them in detail (e.g., data contain potentially identifying or sensitive patient information, data are owned by a third-party organization, etc.) and who has imposed them (e.g., a Research Ethics Committee or Institutional Review Board, etc.). Please also provide contact information for a data access committee, ethics committee, or other institutional body to which data requests may be sent.

*b) If **there are no restrictions,** please upload the minimal anonymized data set necessary to replicate your study findings to a stable, public repository and provide us with the relevant URLs, DOIs, or accession numbers. For a list of recommended repositories, please see https://journals.plos.org/plosone/s/recommended-repositories. You also have the option of uploading the data as Supporting Information files, but we would recommend depositing data directly to a data repository if possible.*

In response to your query, there are actual legal restrictions regarding the 203/2015 Decree of the Catalonian surveillance epidemiological network Data Protection, where individuals who, by virtue of their duties, are involved in the process of notifying notifiable diseases and epidemic outbreaks are subject to the duty of confidentiality in this regard. The body responsible for processing the personal data obtained through the notification systems described in this Decree, and the data processors, must adopt the necessary technical, management, and organizational measures to ensure the confidentiality, security, and integrity of the data, as well as control access to the data from the moment the forms are received by any of the means provided, and ensure their traceability. They must also take the necessary actions to enforce the rights recognized in the personal data protection regulations.

Nevertheless, we have discussed the best way to present the anonymized data with only the information included in the manuscript, and we have decided to upload a simplified dataset to ZENODO, available at DOI: 10.5281/zenodo.13284254. Sensitive information, such as date of birth, has been reduced to the year of birth. The country of birth has been simplified to regions: Spain, Europe, Asia, Africa, North America, and Latin America and the Caribbean. For geolocation, only the ZIP code of residence and the categorization in the Basic Health Area are presented. We believe that with this amount of information, the data presented is sufficiently reproducible. This information is detailed in the manuscript accordingly.

*3. When completing the data availability statement of the submission form, you indicated that you will make your data available on acceptance. We strongly recommen**d all authors decide on a data sharing plan before acceptance**, as the process can be lengthy and hold up publication timelines. Please note that, though access restrictions are acceptable now, your entire data will need to be made freely accessible if your manuscript is accepted for publication. This policy applies to all data except where public deposition would breach compliance with the protocol approved by your research ethics board. If you are unable to adhere to our open data policy, please kindly revise your statement to explain your reasoning and we will seek the editor's input on an exemption. Please be assured that, once you have provided your new statement, the assessment of your exemption will not hold up the peer review process.*

As solved in the previous note, the entire data is now accessible prior to the publication of the results, after a discussed anonymization process.

*4. Please include your full ethics statement in the **'Methods'** section of your manuscript file. In your statement, please include the full name of the IRB or ethics committee who approved or waived your study, as well as whether or not you obtained informed written or verbal consent. If consent was waived for your study, please include this information in your statement as well.*

As presented in the methodology section of the article, and described in previous outbreaks in the city published in PLOS ONE (Forcadell-Diez et al, 2023, DOI: 10.1371/journal.pone.0280614), this outbreak report is exempt from the requirement of approval by an ethics committee because it was conducted under the authority of Article 5.b. of Catalonia's Health Department Decree 203/2015, dated 15 September. This decree established the Epidemiological Surveillance Network and regulates the reporting systems for notifiable diseases and epidemic outbreaks, without the need of IRB aproval prior to conduct any investigation. Participants did not provide informed consent due to the regulatory framework governing of reporting systems for notifiable diseases and epidemic outbreaks. Five cases (0.3% of the sample) involved individuals aged 15-17 years, who required verbal consent from the parents or legal guardians

5. We note that Figure 3 in your submission contain map/satellite images which may be copyrighted. All PLOS content is published under the Creative Commons Attribution License (CC BY 4.0), which means that the manuscript, images, and Supporting Information files will be freely available online, and any third party is permitted to access, download, copy, distribute, and use these materials in any way, even commercially, with proper attribution. For these reasons, we cannot publish previously copyrighted maps or satellite images created using proprietary data, such as Google software (Google Maps, Street View, and Earth). For more information, see our copyright guidelines: http://journals.plos.org/plosone/s/licenses-and-copyright.

*We require you to either (a) **present written permission from the copyright holder to publish these figures specifically under the CC BY 4.0 license**, or (b) remove the figures from your submission:*
a. You may seek permission from the original copyright holder of Figure 3 to publish the content specifically under the CC BY 4.0 license.

*We recommend that you contact the original copyright holder with the Content Permission Form (http://journals.plos.org/plosone/s/file?id=7c09/content-permission-form.pdf) and the following text: "I request permission for the open-access journal PLOS ONE to publish XXX under the Creative Commons Attribution License (CCAL) CC BY 4.0 (http://creativecommons.org/licenses/by/4.0/). Please be aware that this license allows unrestricted use and distribution, even commercially, by third parties. Please reply and provide explicit written permission to publish XXX under a CC BY license and complete the attached form." Please upload the completed Content Permission Form or other proof of granted permissions as an "Other" file with your submission. In the figure caption of the copyrighted figure, please include the following text: **"Reprinted from [ref] under a CC BY license, with permission from [name of publisher], original copyright [original copyright year**]."*

b. If you are unable to obtain permission from the original copyright holder to publish these figures

under the CC BY 4.0 license or if the copyright holder's requirements are incompatible with the CC BY 4.0 license, please either i) remove the figure or ii) supply a replacement figure that complies with the CC BY 4.0 license. Please check copyright information on all replacement figures and update the figure caption with source information. If applicable, please specify in the figure caption text when a figure is similar but not identical to the original image and is therefore for illustrative purposes only. The following resources for replacing copyrighted map figures may be helpful:

USGS National Map Viewer (public domain): http://viewer.nationalmap.gov/viewer/
The Gateway to Astronaut Photography of Earth (public domain): http://eol.jsc.nasa.gov/sseop/clickmap/
Maps at the CIA (public domain): https://www.cia.gov/library/publications/the-world-factbook/index.html and https://www.cia.gov/library/publications/cia-maps-publications/index.html
NASA Earth Observatory (public domain): http://earthobservatory.nasa.gov/
Landsat: http://landsat.visibleearth.nasa.gov/
USGS EROS (Earth Resources Observatory and Science (EROS) Center) (public domain): http://eros.usgs.gov/#
Natural Earth (public domain): http://www.naturalearthdata.com/

We appreciate the comment provided, because our data aims to be free of copyrights. To address this comment, we had to review other images published at PLOS from other authors, as well as maps published by our own institution in PLOS ONE before (Forcadell-Diez et al, 2023; Torres ML et al, 2022). We are completely sure that our presented map is not copyrighted and can be freely distributed under the Creative Commons Attribution License (CC BY 4.0). The images were created with the software QGIS, which is a Free and open-source software, with QGIS maps of Barcelona and other territories previously published in PLOS ONE.

Furthermore, the city layers used are available for free at the portal Carto BCN[1] where formats .shp among others are available. Carto BCN is part of the Open data BCN project[2], which is subject to a CC BY 4.0 license[3], meaning that the information can be reused (copied, adapted, processed, etc.) and distributed on the condition that the source of the data is cited. However, in the case of data types involving third-party participation, such as ours, reuse is facilitated through the Creative Commons Attribution 4.0 International license, No Derivatives (CC BY-ND 4.0).

Some types of information are subject to specific legislation, such as, for example, geographic information, which is covered by the Cartographic Law of Catalonia, or urban planning information, among others. The Barcelona City Council warns that the municipal parcel registry and the urban planning information shown on this website regarding qualifications, planning and management areas, license suspension, and heritage have merely informative and non-normative character. The documents contained on the website comply with the telematic information requirements provided in urban planning regulations. It is mandatory to acknowledge authorship and mention the Barcelona City Council as the source of the datasets, as follows: Data source: Barcelona City Council. Users are encouraged to inform the Barcelona City Council of all projects related to the use of the datasets or

[1] Carto BCN: https://w20.bcn.cat/cartobcn/default.aspx?lang=es
[2] Open Data BCN: https://opendata-ajuntament.barcelona.cat/es
[3] FAQs | Open Data BCN: https://opendata-ajuntament.barcelona.cat/es/faqs/#collapseEighteen

that derive from them, so that they can be publicly displayed with the aim of promoting public sector information reuse policies. Additionally, the Barcelona City Council may request statistical data from users regarding products or services generated from the reused information, in order to understand the real use of the information reuse and to improve the catalog of available data.

Because of that we believe that our maps are free of any copyright, and are suitable to be presented in your journal, although some modifications have been presented in the statiscital analyses and in the description of the figures: "Maps were created with QGIS Geographic Information System version 3.4.8-Madeira, and all content is licensed under the Creative Commons Attribution–Share Alike 3.0 license (CC BY–SA), available at (https://qgis.org). In addition, geographical materials used for creating the map (e.g., shape file) were supported by Carto BCN from the Open data BCN project, Barcelona City Council and MPOX data from the Epidemiology service registers. The authors specify that this figure is licensed under CC BY 4.0."

*6. Please include **captions for your Supporting Information files** at the end of your manuscript, and update any in-text citations to match accordingly. Please see our Supporting Information guidelines for more information: http://journals.plos.org/plosone/s/supporting-information.*

Thanks for the notification. All the supporting information files are included according to guidelines, being modified from "Supplementary 1 or 2" to "S1 Appendix 1 and 2", with added captions at the end of the manuscript. We only named one file as S1, because in the same PDF are presented Appendix 1 and 2.

Reviewers' comments:

1. Is the manuscript technically sound, and do the data support the conclusions?

The manuscript must describe a technically sound piece of scientific research with data that supports the conclusions. Experiments must have been conducted rigorously, with appropriate controls, replication, and sample sizes. The conclusions must be drawn appropriately based on the data presented.

Reviewer #1: Yes

Reviewer #2: No

Methodology has been updated, with changes in the inclusion and exclusion criteria, the statistical analyses developed and the source of some of the presented images.

2. Has the statistical analysis been performed appropriately and rigorously?

Reviewer #1: Yes

Reviewer #2: No

We believe that our data has been extensively analyzed, however, we understand that some information could be improved, such as immunosuppression status, prior vaccination status for orthopoxvirus, and comprehensive clinical and laboratory findings. We have updated the results with the available information previously non presented.

3. Have the authors made all data underlying the findings in their manuscript fully available?

The PLOS Data policy requires authors to make all data underlying the findings described in their manuscript fully available without restriction, with rare exception (please refer to the Data Availability Statement in the manuscript PDF file). The data should be provided as part of the manuscript or its supporting information, or deposited to a public repository. For example, in addition to summary statistics, the data points behind means, medians and variance measures should be available. If there are restrictions on publicly sharing data—e.g. participant privacy or use of data from a third party—those must be specified.

Reviewer #1: Yes

Reviewer #2: No

Anonymized data has been deposited in a public repository.

4. Is the manuscript presented in an intelligible fashion and written in standard English?

PLOS ONE does not copyedit accepted manuscripts, so the language in submitted articles must be clear, correct, and unambiguous. Any typographical or grammatical errors should be corrected at revision, so please note any specific errors here.

Reviewer #1: Yes

Reviewer #2: No

Although a native revision was made before the submission, a new one was conducted. Improvements in English language are marked in yellow highlight.

5. Review Comments to the Author

Please use the space provided to explain your answers to the questions above. You may also include additional comments for the author, including concerns about dual publication, research ethics, or publication ethics. (Please upload your review as an attachment if it exceeds 20,000 characters)

Reviewer #1: This publication on an emerging infectious disease is quite important to its global preventive measures. The experience of the authors in the identification of the most susceptible group that are prone to Mpox will inform public health intervention by health leaders globally.

We appreciate the kind words, and hope that our results, as you comment, could help the develop of public health interventions globally. The learings of health comunication presented in our r

Reviewer #2: The manuscript addresses an interesting topic but has several issues that need to be addressed:

1. The structure does not follow a typical descriptive study format. Key sections like inclusion and exclusion criteria for notified cases, as well as statistical analysis, are missing or inadequately described.

We appreciate the comment provided and have made efforts to improve the outbreak manuscript format to follow a descriptive study. In that line, inclusion and exclusion criteria have been added as a single paragraph, while statistical analyses have been presented separately of source of information.

2. Essential descriptive data, such as the proportion of immunosuppression status, prior vaccination status for orthopoxvirus, and comprehensive clinical and laboratory findings, are not well-presented in the results section. Figures, charts, and tables lack adequate text descriptions.

Changes have been made to all the commented issues with the available information. Also, the figures, charts and tables descriptions have been improved. However, laboratory findings were not presented, due to the high amount of missing data, with individuals who received both a generic POX and specific MPOX testing, while others who did not have information. We believe, reviewing similar literature, that the absence of information does not affect the outbreak's description.

3. The authors label the study as the "first report" of MPOX in the region, despite existing research reporting MPOX in the same area (e.g., https://doi.org/10.1093/ofid/ofae105).

This is an important debate because we can confidently state that our complete data is not fully represented in the results presented in this article. For instance, a single city has reported more diagnoses than the 52 notifying centers combined. While we acknowledge the contributions of other authors, their study reports 1,472 cases, whereas ours presents 1,684.

The study by Ramirez-Olivencia et al., a descriptive analysis of a series of cases, emphasizes that the majority of cases were reported in Madrid, Catalonia, and Andalusia. In contrast, our analysis, which is an outbreak report, focuses specifically on a single city—Barcelona (part of Catalonia)—and includes time-specific measures and preventive actions. We observed that while two centers in Barcelona may have reported cases to the study, they likely did not account for the total number of cases from each center. Additionally, it is probable that most cases included in the study were reported from Badalona, a city close to Barcelona, where the Hospital Can Ruti is located, and where some of the study's authors practice.

4. The abstract is missing a background section, and the ***original*** *source of Figure 1 is not cited.*

Changes have been made in order to improve the quality of the abstract, as well as the original source of figure 1.

[NOTE: If reviewer comments were submitted as an attachment file, they will be attached to this email and accessible via the submission site. Please log into your account, locate the manuscript record, and check for the action link "View Attachments". If this link does not appear, there are no attachment files.]

No more comments were found from the reviewers.

While revising your submission, please upload your figure files to the Preflight Analysis and Conversion Engine (PACE) digital diagnostic tool, https://pacev2.apexcovantage.com/. PACE helps ensure that figures meet PLOS requirements. To use PACE, you must first register as a user. Registration is free. Then, login and navigate to the UPLOAD tab, where you will find detailed instructions on how to use the tool. If you encounter any issues or have any questions when using PACE, please email PLOS at <a href="mailto:figures@plos.org">figures@plos.org. Please note that Supporting Information files do not need this step.

Figures were uploaded to PACE and guidelines were reviewed, so improved figures are presented.
