# Supplementary material for: A new STI in the city: MPOX in Barcelona. First outbreak (5/2022-5/2023) and subsequent resurgence
Source: PLoS One. 2025 Jan 16;20(1):e0296141. doi: 10.1371/journal.pone.0296141 (PMC11737761; doi:10.1371/journal.pone.0296141)
Supplement: Attachment — Submitted filename: Response to Reviewers.docx [file pone.0296141.s004.docx]

**Response to Reviewers**

1. Please review your reference list to ensure that it is complete and correct. If you have cited papers that have been retracted, please include the rationale for doing so in the manuscript text, or remove these references and replace them with relevant current references. Any changes to the reference list should be mentioned in the rebuttal letter that accompanies your revised manuscript. If you need to cite a retracted article, indicate the article’s retracted status in the References list and also include a citation and full reference for the retraction notice.

All references were revised and are correct. Only one reference has been added, about a WHO recommendation made in 1980: “*Global Commission for the Certification of Smallpox Eradication & World Health Organization. The global eradication of smallpox : final report of the Global Commission for the Certification of Smallpox Eradication [Internet]. Geneva; 1979 Dec [cited 2024 Nov 8]. Available from: https://iris.who.int/handle/10665/39253*”

2. Additional Editor Comments:

   Dear Authors
   Please revise the draft as per reviewer suggestions.
   [Note: HTML markup is below. Please do not edit.]

   Reviewers' comments:
   Reviewer's Responses to Questions
3. **Comments to the Author**

   1. If the authors have adequately addressed your comments raised in a previous round of review and you feel that this manuscript is now acceptable for publication, you may indicate that here to bypass the “Comments to the Author” section, enter your conflict of interest statement in the “Confidential to Editor” section, and submit your "Accept" recommendation.

Reviewer #1: All comments have been addressed

Reviewer #2: All comments have been addressed

2. Is the manuscript technically sound, and do the data support the conclusions?
The manuscript must describe a technically sound piece of scientific research with data that supports the conclusions. Experiments must have been conducted rigorously, with appropriate controls, replication, and sample sizes. The conclusions must be drawn appropriately based on the data presented.

Reviewer #1: Yes

Reviewer #2: Yes

3. Has the statistical analysis been performed appropriately and rigorously?

Reviewer #1: Yes

Reviewer #2: Yes

4. Have the authors made all data underlying the findings in their manuscript fully available?

All the data is available at DOI: 10.5281/zenodo.13284254

The [PLOS Data policy](http://www.plosone.org/static/policies.action#sharing) requires authors to make all data underlying the findings described in their manuscript fully available without restriction, with rare exception (please refer to the Data Availability Statement in the manuscript PDF file). The data should be provided as part of the manuscript or its supporting information, or deposited to a public repository. For example, in addition to summary statistics, the data points behind means, medians and variance measures should be available. If there are restrictions on publicly sharing data—e.g. participant privacy or use of data from a third party—those must be specified.

Reviewer #1: Yes

Reviewer #2: Yes

5. Is the manuscript presented in an intelligible fashion and written in standard English?

PLOS ONE does not copyedit accepted manuscripts, so the language in submitted articles must be clear, correct, and unambiguous. Any typographical or grammatical errors should be corrected at revision, so please note any specific errors here.

Reviewer #1: Yes

**Reviewer #2: No**

A fully revised version by native english has been submitted.

6. Review Comments to the Author
Please use the space provided to explain your answers to the questions above. You may also include additional comments for the author, including concerns about dual publication, research ethics, or publication ethics. (Please upload your review as an attachment if it exceeds 20,000 characters)

**Reviewer #1: The authors have adequately addressed my comments on statistical analysis in the revised version of the manuscript.**

Answer: Thank you so much for the revision.

**Reviewer #2: The manuscript needs to be thoroughly proofread for typos and language improvements. For instance, 'COVID-19' should be written in all caps throughout the text. All changes made by the authors should be highlighted in the manuscript.**

Answer: Thank you so much as well, all changes are highlighted.

PLOS authors have the option to publish the peer review history of their article ([what does this mean?](https://journals.plos.org/plosone/s/editorial-and-peer-review-process#loc-peer-review-history)). If published, this will include your full peer review and any attached files. If you choose “no”, your identity will remain anonymous but your review may still be made public.
**Do you want your identity to be public for this peer review?** For information about this choice, including consent withdrawal, please see our [Privacy Policy](https://www.plos.org/privacy-policy).

Reviewer #1: **Yes:** Ado G Abubakar

Reviewer #2: **Yes:** Zahra Heydarifard
